# Postinfarction ventricular septal defect: A new surgical option without left ventriculotomy

**DOI:** 10.1016/j.xjtc.2023.03.011

**Published:** 2023-03-31

**Authors:** Mario Torre, Antonio Longobardi, Maria Giovanna Vassallo, Guido Oppido, Enrico Coscioni

**Affiliations:** a Cardiac Surgery Division, AOU San Leonardo, Salerno, Italy; b Division of Pediatric Cardiac Surgery, Ospedale Monaldi, Naples, Italy

**Figure:**
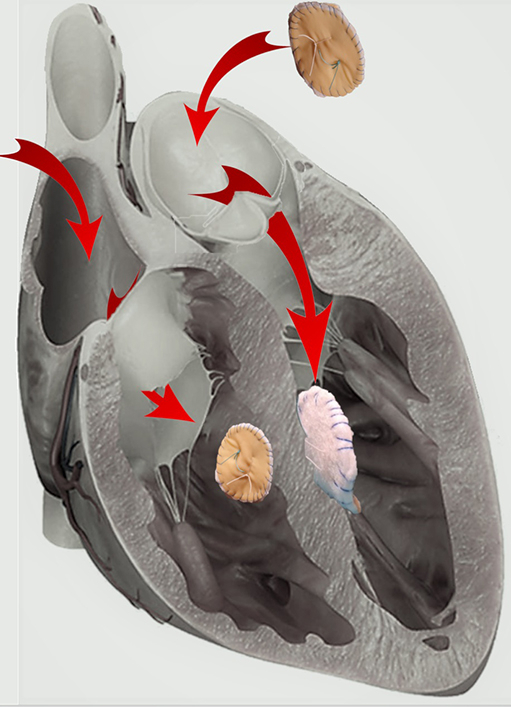
PVSD closure parachuting 2 composite handmade patches through the AV and the TV.

**Central Message**

Successful PSVD closure parachuting 2 composite handmade patches through the AV and the TV, without LVT. It is feasible, simple, and provides good results at 4-months follow-up.

Postinfarction ventricular septal defect (PVSD) is a rare complication of myocardial infarction (MI) with ominous prognosis.^1^ Although left ventriculotomy (LVT) and infarct exclusion technique^2^ or infarctectomy^3^ are considered standard approaches, posterobasal PVSDs are challenging and have higher operative mortality risk. We report the case of a 66-year-old patient with an ST-elevation MI and 1.4 cm PSVD, who underwent urgent surgery. He was successfully treated with a novel, simple closure, using 2 handmade patches parachuted through the aortic valve (AV) and the tricuspid valve (TV), without the need to perform LVT.

## Methods

The patient gave written informed consent for publication of study data. Institutional review board approval was not required.

## Technique

Two composite patches were made before cardiopulmonary bypass (CPB) institution using 2 polytetrafluoroethylene layers and a pericardial disk (Video 1). CPB was established and myocardial protection was achieved. Transversal aortotomy and right atriotomy were performed to have direct view and access to the septum through the AV and the TV. It is easier to start the inspection from the right aspect of the septum, pulling upward the TV and right atrium. Once the PVSD was found, a silicon surgical loop was placed as a marker through the AV, the PSVD, and the TV using a right-angle forceps either passing through the AV or the TV.

**Figure 1:**
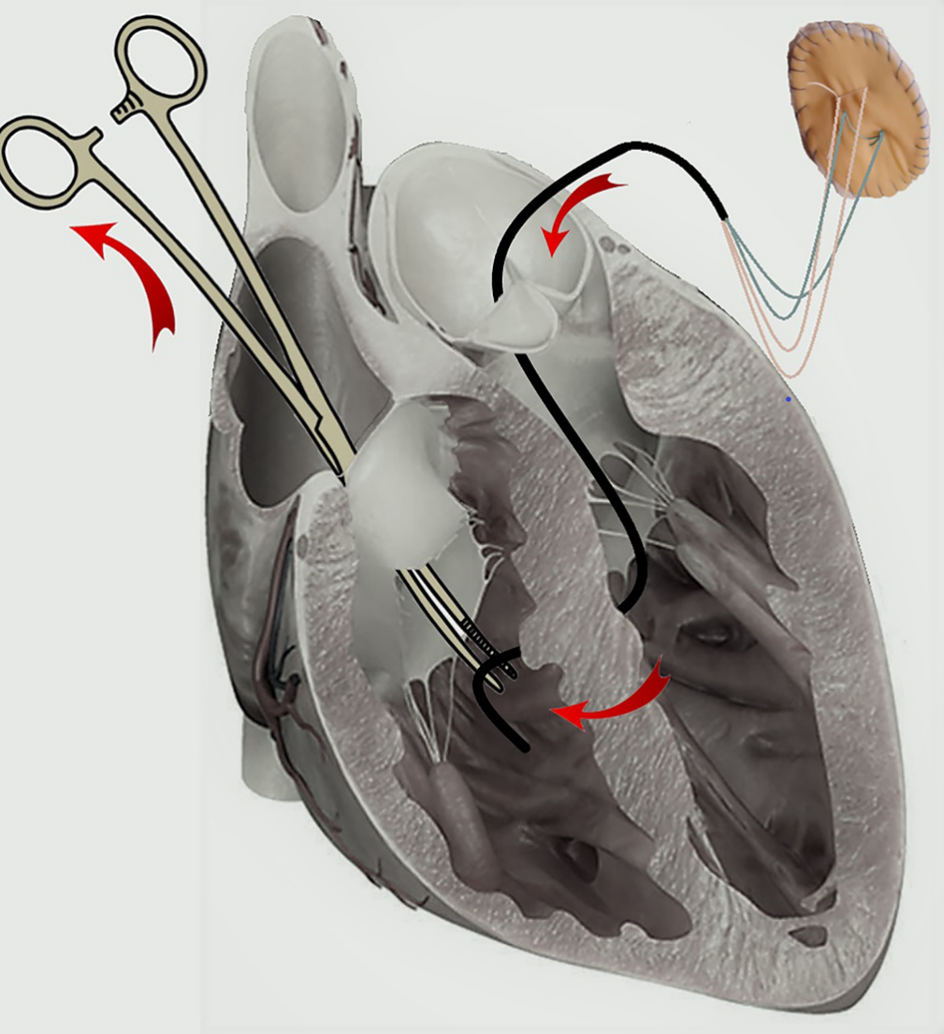
A right angle or a dissector forceps is passed through the tricuspid valve (TV) and the postinfarction ventricular septal defect (PSVD), until it is visible through the aortic valve (AV), to grasp a silicon surgical loop, placed through the AV, the PSVD, and the TV. The loop can also be placed by passing the forceps through the AV, which is a safer access to avoid mitral subvalvolar apparatus. Ethibon 2–0 sutures of the left patch are then knotted to the aortic extremity of the loop, so that by pulling out the tricuspid extremity of the loop, the patch is parachuted down in the left ventricle—with its polytetrafluoroethylene layer against the septum—through the AV, whereas the Ethibon 2–0 sutures are passed across the PVSD, the TV, and out of the right atrium.

**Figure 2:**
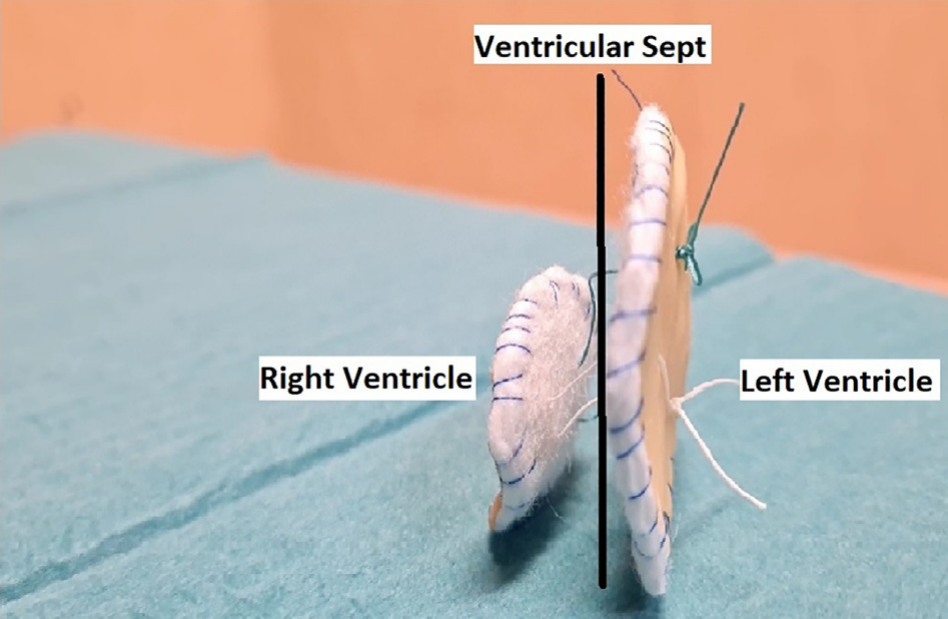
The pericardial layers of the 2 patches must face the left and the right ventricular cavities, whereas the polytetrafluoroethylene layers adhere to the interventricular septum. Generally—from pediatric cardiac surgery experience—there is no need to anchor the patches. Theoretically there might be the risk of patch migration, but it should not happen if they are well sized. It is very difficult for the left-side patch to migrates from left to right because it is oversized, but it may be useful to anchor the right 1 to the right ventricle trabeculae, which are generally not involved in myocardial infarction.

Ethibon 2–0 sutures were knotted to the aortic extremity of the surgical loop and then the bigger left-side patch was parachuted against the septum by pulling out the tricuspid extremity of the loop (Figure 1). Then, after the loop was cut off, the same Ethibon 2–0 sutures were passed through the smaller right-side patch, which in turn was parachuted down in the right ventricle and knotted, tightening together the 2 patches (Figure 2 and Video 2). The mitral valve and TV were assessed directly and with transoesophageal echocardiography to check for any interference. CPB time was 65 minutes and crossclamp time was 51 minutes.

## Discussion

Due to its unfavorable position and the unavoidable extension of the MI to the right ventricle, posterior PSVD is associated with increased risk of operative mortality.^4^ Several techniques have been developed: infarctectomy and infarct exclusion techniques are standard for PSVD repair, but surgically demanding. Compared with those techniques, our new approach is technically simpler—it only requires an aortotomy and a right atriotomy—is time-saving (shorter CPB and crossclamp times), myocardial tissue-saving (avoiding LVT and longer crossclamping, we reduce further myocardial impairment and deterioration of left ventricular function). Moreover, it eases bleeding control.

Our patch technique takes inspiration from PDSV percutaneous closure, overcoming some limitations and drawbacks. Firstly, patch sizing can be more tailored, using the biggest patch possible to ensure closure of complex or irregular defects, directly checking any damage and interference with the mitral valve and TV. Moreover, the absence of a waist between the disks allows a perfect allocation across the septum, avoiding device displacement and exerting no pressure on necrotic tissues due to nickel titanium's expanding radial force. Finally, the right patch can be also anchored with 5–0 polypropylene single stiches to avoid displacement or TV interference. Hybrid approaches may be an interesting strategy.

Regarding surgical timing, the ideal timing of repair remains controversial. Better outcomes are reported with delayed closure, but these results might be confounded by selection and survival bias. In this case, the heart team decided to perform urgent surgery, taking advantage of the hemodynamic stability. No more benefit was expected with watchful waiting. Furthermore, this approach can also be used in early/urgent surgery; it is not necessary to wait for the infarcted myocardial tissue to consolidate.

## Conclusions

This new technique is feasible, simple, and provides good short-term results (Video 3). Theoretically, all types of PSVD, in every anatomical position, might be addressed with this technique; it could be the first-line approach, and, in case of failure, it can be quickly repeated adding a second or third patch, or converted into a standard approach.

## Supplementary Data

Video 1 Each patch is made by sewing together an autologous/bovine pericardial disc with 2 polytetrafluoroethylene layers with 5–0 polypropylene suture. The polytetrafluoroethylene disks make the patch thicker, but still flexible, whereas the pericardial layer is a less thrombogenic surface. Measures are transoesophageal echocardiography-based: the patch should be 2 to 3 cm larger than the postinfarction ventricular septal defect (PSVD) diameter and the left 1 should be bigger than the right 1 due to different pressure they deal with and to avoid any interference with the septal leaflet of the tricuspid valve. Two Ethibon 2–0 mattress sutures are passed through the central part of the left-side patch, with large bites (5-7 mm) in button-seam fashion. Then Ethibon needles are cut off. A PSVD cutoff has not been determined because this is the first case. However, it might be effective also in quite large PVSD, because the patches can be tailored on PVSD size and, in case of failure, more than 1 patch can be used. Video available at: https://www.jtcvs.org/article/S2666-2507(23)00109-8/fulltext.

Video 2 By pulling out the tricuspid extremity of the surgical loop, the bigger left-side patch is parachuted down in the left ventricle, against the postinfarction ventricular septal defect, through the aortic valve, while the Ethibon 3–0 sutures are passed across the septum and the tricuspid valve, out of the right atrium. Then, the surgical loop is cut off and, using a hollow needle, Ethibon 3–0 sutures are passed through the central part of the smaller right-side patch. The patch is then parachuted down in the right ventricle by finger pressure and then knotted, tightening together the 2 patches. Video available at: https://www.jtcvs.org/article/S2666-2507(23)00109-8/fulltext.

Video 3 No residual left-to-right shunting at the postrepair transoesophageal echocardiography (TEE) evaluation. This result was confirmed at the echocardiographic follow-up and clearly showed by a 4-month angio-computed tomography scan. The patches should prevent any shunt, for several reasons. Firstly, the intraventricular pressures push them against the septum, and—to create a larger adhesion area—the central Ethibon stiches must be placed not right in the middle of the patches, but a little bit outer. Secondly, the left-side patch must be always bigger than the right-side one. Moreover, the 2 patches are rigid enough to maintain their structural shape, but still quite flexible to adapt to the muscular surface, as in the case of a lack of septal rim in an extremely postero-basal postinfarction ventricular septal defect. A residual shunt might be present, but it should be trivial and not hemodynamically significant: It can be assessed intraoperatively with TEE and by measuring oxygen saturation in pulmonary artery and right atrium blood samples. Video available at: https://www.jtcvs.org/article/S2666-2507(23)00109-8/fulltext.
