# Supplementary figures and images for: Postinfarction ventricular septal defect: A new surgical option without left ventriculotomy
Source: JTCVS Tech. 2023 Mar 31;19:49–51. doi: 10.1016/j.xjtc.2023.03.011 (PMC10267962; doi:10.1016/j.xjtc.2023.03.011)

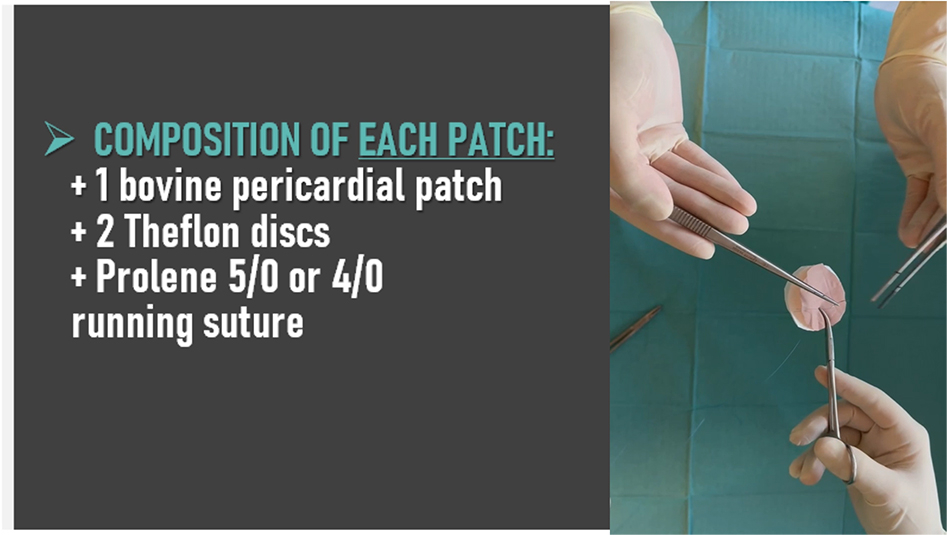

Supplement: Video 1 — Each patch is made by sewing together an autologous/bovine pericardial disc with 2 polytetrafluoroethylene layers with 5–0 polypropylene suture. The polytetrafluoroethylene disks make the patch thicker, but still flexible, whereas the pericardial layer is a less thrombogenic surface. Measures are transoesophageal echocardiography-based: the patch should be 2 to 3 cm larger than the postinfarction ventricular septal defect (PSVD) diameter and the left 1 should be bigger than the right 1 due to different pressure they deal with and to avoid any interference with the septal leaflet of the tricuspid valve. Two Ethibon 2–0 mattress sutures are passed through the central part of the left-side patch, with large bites (5-7 mm) in button-seam fashion. Then Ethibon needles are cut off. A PSVD cutoff has not been determined because this is the first case. However, it might be effective also in quite large PVSD, because the patches can be tailored on PVSD size and, in case of failure, more than 1 patch can be used. Video available at: https://www.jtcvs.org/article/S2666-2507(23)00109-8/fulltext. [file fx2.jpg]

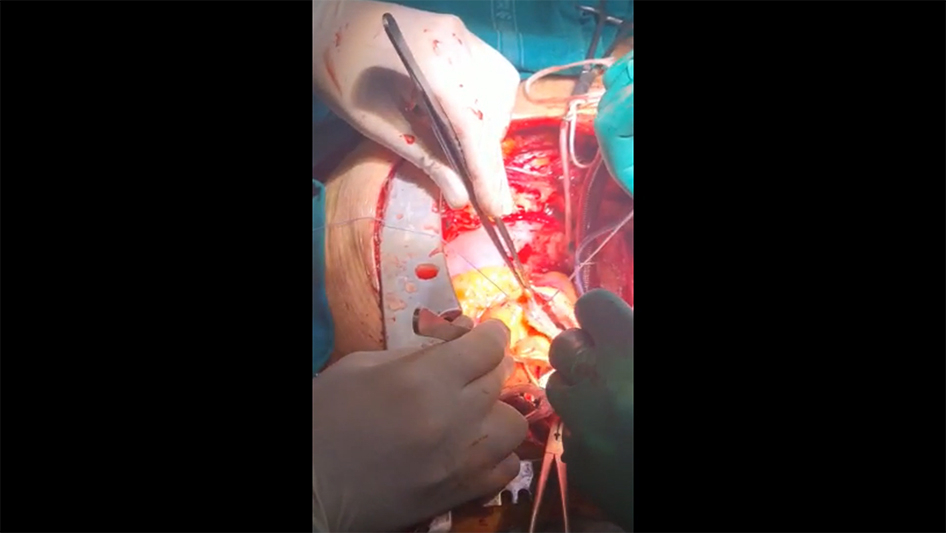

Supplement: Video 2 — By pulling out the tricuspid extremity of the surgical loop, the bigger left-side patch is parachuted down in the left ventricle, against the postinfarction ventricular septal defect, through the aortic valve, while the Ethibon 3–0 sutures are passed across the septum and the tricuspid valve, out of the right atrium. Then, the surgical loop is cut off and, using a hollow needle, Ethibon 3–0 sutures are passed through the central part of the smaller right-side patch. The patch is then parachuted down in the right ventricle by finger pressure and then knotted, tightening together the 2 patches. Video available at: https://www.jtcvs.org/article/S2666-2507(23)00109-8/fulltext. [file fx3.jpg]

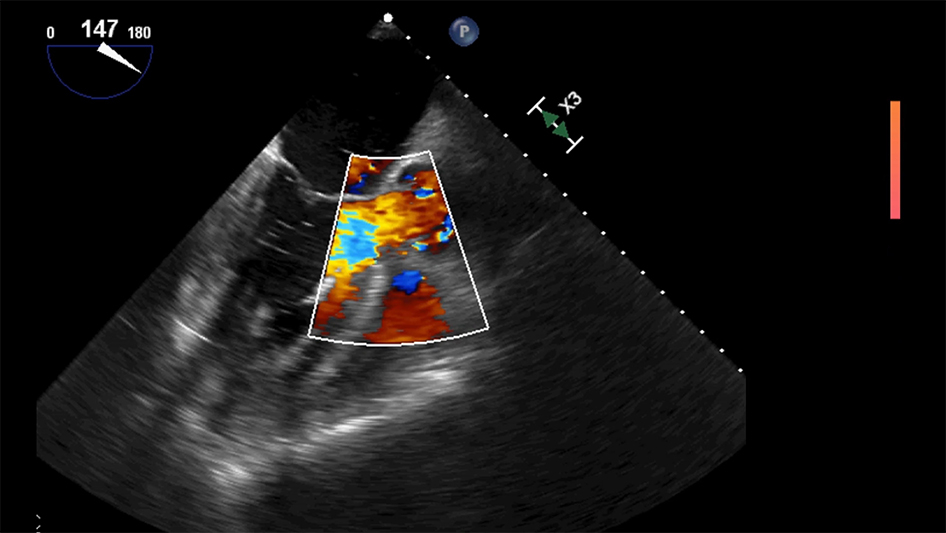

Supplement: Video 3 — No residual left-to-right shunting at the postrepair transoesophageal echocardiography (TEE) evaluation. This result was confirmed at the echocardiographic follow-up and clearly showed by a 4-month angio-computed tomography scan. The patches should prevent any shunt, for several reasons. Firstly, the intraventricular pressures push them against the septum, and—to create a larger adhesion area—the central Ethibon stiches must be placed not right in the middle of the patches, but a little bit outer. Secondly, the left-side patch must be always bigger than the right-side one. Moreover, the 2 patches are rigid enough to maintain their structural shape, but still quite flexible to adapt to the muscular surface, as in the case of a lack of septal rim in an extremely postero-basal postinfarction ventricular septal defect. A residual shunt might be present, but it should be trivial and not hemodynamically significant: It can be assessed intraoperatively with TEE and by measuring oxygen saturation in pulmonary artery and right atrium blood samples. Video available at: https://www.jtcvs.org/article/S2666-2507(23)00109-8/fulltext. [file fx4.jpg]
